# Recruiting and Engaging Older Men in Evidence-Based Health Promotion Programs: Perspectives on Barriers and Strategies

**DOI:** 10.1155/2016/8981435

**Published:** 2016-06-05

**Authors:** Chelsie Anderson, Laura R. Seff, Anamika Batra, Chintan Bhatt, Richard C. Palmer

**Affiliations:** Florida International University, Miami, FL 33199, USA

## Abstract

Evidence-based health promotion programs are effective at reducing health risks and healthcare costs among older adults, but few men participate in the programs. This mixed methods study aimed to gain insight into the barriers to recruiting and engaging older men in evidence-based health promotion programs offered by the Healthy Aging Regional Collaborative of South Florida (HARC). Fourteen program coordinators participated in a focus group to identify barriers and strategies to improve male participation, and 49 instructors participated in a survey to triangulate the findings. Themes among barriers to male participation included women outnumbering men in the implementation sites and programs, conflict between male gender roles and the programs, and preference for other activities. Themes among strategies included public support of programs by male community leaders, program advertisements featuring males, and adapting program content. Survey results supported themes identified in the focus group. Nearly 78% of the survey respondents agreed that the perception of exercise programs as feminine was a barrier and over 90% of the survey respondents believed program advertisements featuring men would increase male participation. Findings indicate that health promotion programs and recruiting strategies need to be tailored to the unique needs and preferences of older men to improve participation.

## 1. Introduction

The number of adults aged 65 and older in the United States is projected to increase to 71 million by 2030 [1]. Eighty percent of older adults have at least one chronic disease and 50% have at least two chronic diseases [1]. Treatment of chronic conditions for older adults accounts for 66% of the healthcare budget in the United States [2]. Impairment and disability are also common and costly among older adults [3]. The chronic disease burden, disability, and healthcare costs associated with an aging population have made the health status of older adults a top priority for the Centers for Disease Control and Prevention (CDC), the Administration on Aging (AoA), and Medicare.

To ensure that limited resources for improving the health status of older adults are used effectively and efficiently, policymakers, funders, and members of the aging networks are increasingly calling for the use of evidence-based health promotion (EBHP) programs. Research supports the effectiveness of EBHP programs in improving health behaviors and health outcomes among older adults of different ethnicities and in different communities [4–9]. Researchers have also demonstrated that EBHP programs reduce healthcare utilization and healthcare costs [6, 7, 10].

Federal law now requires health promotion and disease prevention programs to be evidence-based in order to be eligible for Older Americans Act Title IIID funding [11]. In 2014, 43 programs that underwent the Older Americans Act Title IIID submission process met the highest-level criteria for evidence-based programs and include programs such as the Stanford Self-Management programs, EnhanceFitness, and Matter of Balance [12]. Evidence-based programs are supported as being effective; however, members of the intended population must participate in the programs in order to benefit from them.

A variety of factors may influence an older individual's willingness to participate in health promotion programs. Cultural beliefs, attitudes about health promotion, gender roles, age-related issues, and mistrust of research have been reported as reasons why older Korean Americans do not participate in health promotion programs [13]. Fatalism, underestimation of risk, poor self-efficacy, and stigma associated with programs for older people were identified as barriers to participation in fall prevention programs [14]. Research with participants, nonparticipants, and administrators of a health promotion program designed to reduce cardiovascular disease in older adults suggests that time constraints, transportation, lack of information about the programs, and lack of comfort are barriers to participation [15, 16]. Focus groups conducted at a senior wellness center revealed that social support, interpersonal engagement, and programs tailored to individual needs, interests, and limitations facilitate participation in community-based health promotion programs [17]. A study of older adults who declined to join or joined an exercise program revealed that those who joined were socially inclined and desired accountability while those who declined to join reported already getting enough exercise, lack of motivation, and lack of affiliation with people in the program [18].

A significant issue related to older adult participation in EBHP programs is that men are often underrepresented [4, 8, 19–21]. Given that males also suffer higher rates of most illnesses than females and have higher healthcare costs than females from the age of 60 to approximately the age of 90, efforts are needed to ensure that older men participate in EBHP programs [22]. While it is clear from the literature that men and women differ in the frequency of many health behaviors that may contribute to gender disparities in healthcare costs, the reasons why men are less likely than women to engage in health behaviors such as participating in EBHP programs are poorly understood [23].

Research suggests that a one-size-fits-all approach to male health promotion will not be successful because men are a heterogeneous group with varying health information needs and preferences [24, 25]. There is a lack of research addressing the participation of older men in health promotion programs. The present study aimed to address this gap by examining program coordinator and instructor perspectives on low male participation in EBHP programs offered by the Healthy Aging Regional Collaborative of South Florida (HARC). Males represented 44% of the population aged 60 and older in counties where HARC offered programs, but males represented only 19% of HARC participants [26]. As program implementers, HARC coordinators and instructors are responsible for recruiting and engaging participants and thus were selected to participate in this study to identify barriers to recruiting and engaging older male participants and to identify strategies to increase male participation.

## 2. Method

### 2.1. Setting

The Health Foundation of South Florida established the Healthy Aging Regional Collaborative (HARC) in 2008 to disseminate evidence-based health promotion programs in Broward, Miami-Dade, and Monroe counties. Programs were selected to address physical inactivity, fall prevention, and chronic conditions among older adults. The HARC programs included EnhanceFitness (EF), Chronic Disease Self-Management (CDSMP), Tomando Control de su Salud (TCS), Diabetes Self-Management (DSMP), Manejo Personal de la Diabetes (MDP), Matter of Balance (MOB), and Asunto de Equilibrio (ADE). HARC member agencies implemented one or more of the evidence-based programs listed to meet the needs of the older adults they served. This study was approved by the Institutional Review Board at Florida International University.

### 2.2. Participants

Program coordinators and instructors of HARC programs were included in this study to learn why older men were not participating in HARC programs. Program coordinators and instructors were chosen as study participants because they have extensive experience working with program participants and are knowledgeable about the feasibility of potential strategies to increase participation. Each HARC agency selected a program coordinator or multiple program coordinators to manage the implementation of one or more evidence-based programs. Twenty program coordinators representing the 14 HARC member agencies in 2013 were invited by email to participate in a focus group. The 14 program coordinators who participated in the focus group (70% participation rate) represented 11 of the 14 member agencies.

The agencies represented in the focus group included area agencies on aging, nonprofits, healthcare organizations, recreation organizations, and social service agencies. All of the coordinators had managed the HARC programs for multiple years at the time of the study. Program coordinators were responsible for recruiting program participants, so they have knowledge about individuals who participated and individuals who were informed about the programs but decided not to participate.

Program instructors were selected by the HARC agencies and participated in instructor training according to program requirements. HARC program instructors had professional experience in a wide range of fields and facilitated the programs on a part-time or volunteer basis. Program instructors interacted closely with the participants in each program session and were likely to develop a sense of the barriers to participation based on observations and feedback from participants. The 269 program instructors who taught one or more HARC workshops between 2008 and 2013 were invited by email to participate in an online survey. Instructors who were also program coordinators were excluded from the survey. Instructors were sent two reminder emails in an effort to improve the response rate. Forty-nine instructors responded to the survey (18% response rate). Thirty-nine of the respondents (80%) were female and 10 were male (20%). Respondents and nonrespondents did not differ in gender.

### 2.3. Data Collection

This mixed methods study used a focus group to elicit information from HARC program coordinators about the most important barriers and strategies for male participation in HARC programs and to develop a survey about male participation that was subsequently administered to HARC instructors to triangulate findings. The focus group was moderated by a member of the HARC local evaluation team and lasted approximately two hours.

To identify barriers to male participation, the moderator first asked coordinators to provide reasons why more men do not participate in HARC programs and why it is difficult to recruit male participants. The moderator then asked the coordinators to identify strategies that might be effective in increasing male interest and participation in HARC programs. The moderator followed up the initial questions with probes to collect additional information. The moderator documented the ideas reported on an interactive whiteboard grid so that all participants could see the responses as they were given and clarify or expand upon the responses if desired. A note taker from the HARC local evaluation team typed comments from the discussion in an electronic document during the session.

After completing the analysis of the focus group comments, a questionnaire was developed in order to examine instructor perspectives on male participation and to triangulate findings. Major themes from the focus group discussion, interesting but infrequent coordinator comments from the focus group, and participation barriers described in the literature were developed into survey items to examine whether HARC instructors agreed that the factors mentioned by coordinators and existing research were barriers to participation for HARC program participants and whether the suggested strategies were likely to be successful with HARC program participants. HARC instructors received an email inviting them to participate in an online survey. The email included an explanation of the purpose of the study, instructions for accessing the survey questionnaire, a statement explaining that participation was voluntary, and a description of the confidentiality of responses. If participants did not complete the survey within two weeks of receiving the first email, they received two reminder emails spaced one week apart.

The questionnaire was administered using Qualtrics online software and included items that assessed barriers to participation and strategies to increase participation. Items regarding barriers required respondents to rate their level of agreement with several possible explanations for nonparticipation by older males and several statements about their perceptions of male program participants. Items regarding strategies required respondents to indicate the likelihood that each of several potential strategies would be successful in increasing male participation in HARC programs. Open-ended questions asked respondents to describe any barriers to male participation in HARC programs and to list any strategies to increase male participation in HARC programs.

### 2.4. Data Analysis

The focus group data were analyzed by two members of the HARC local evaluation team who collaboratively organized statements from the SMART Board grids and the discussion document into common topics and summarized the data. Descriptive statistics were calculated for the fixed-choice survey questions. In the analysis, ratings of “strongly disagree” and “disagree” were collapsed and ratings of “agree” and “strongly agree” were collapsed. Similarly, ratings of “very unlikely” and “unlikely” were collapsed and ratings of “likely” and “very likely” were collapsed.

Thematic analysis was used to examine the responses to the open-ended survey questions. Thematic analysis involves familiarizing oneself with the dataset, generating codes, combining the codes into themes, and reviewing and refining the themes [27]. The responses to the open-ended survey questions were downloaded from Qualtrics. A member of the evaluation team assigned open codes to words, phrases, or sentences. A second member of the evaluation team validated the codes and then worked collaboratively with the first coder to group the codes into themes.

## 3. Results 

### 3.1. Focus Group

When asked to provide reasons why it is difficult to recruit men in HARC programs, coordinators expressed that older men may not be comfortable in activities where most participants are women and that attention to health in general is not consistent with male gender roles. Coordinators also reported that health promotion programs are not a part of the senior male culture, there are fewer men at senior sites where coordinators recruit participants, social programs are not appealing to men, and there are too many women instructors. Coordinators also believed that men view EnhanceFitness (EF) as an aerobics class and would prefer other physical activities.

When asked to provide a list of strategies to increase male interest and participation in HARC programs, coordinators suggested getting male community leaders to support the programs, producing advertisements showing men, encouraging women to bring men to the workshops, and providing meaningful incentives. Coordinators also suggested encouraging instructors to do less dancing in EF and hiring more male instructors.

### 3.2. Survey

#### 3.2.1. Agreement with Barriers to Male Participation and Men's Beliefs

When asked in the online survey whether older men's viewing exercise programs as aerobics classes for females was a barrier to participation, nearly 78% of the instructors agreed (Table 1). Similarly, nearly 74% of the instructors agreed that machismo or cultural beliefs prevent older men from participating and that older males think personal problems should not be discussed. More than 80% of the instructors disagreed with statements that older males are uncomfortable with female instructors and that older males think they will not be respected if they participate in health promotion programs.

When asked in the online survey to rate their level of agreement with statements regarding men's beliefs and participation, almost 96% of the respondents agreed that men are more likely to participate if they see other men in the group and 87% agreed that men are more likely to participate if they come with their spouse or partner (Table 2). Over 74% of the respondents disagreed that men prefer male instructors.

#### 3.2.2. Descriptions of Barriers to Male Participation

Four themes emerged from open-ended items that asked instructors to describe barriers to male participation. Instructors expressed that more women attend health promotion classes, so men feel outnumbered by women if they do attend. Male gender roles became another apparent theme. One respondent said, “traditionally, females attend to the family's health issues,” and another said, “older males like to feel independent, self-sufficient, and knowledgeable. I think they believe attending one of the classes will indicate the opposite.” Respondents also expressed that men are often not comfortable seeking help or engaging in conversations about health issues.

Another common theme was that men prefer to do other activities rather than attending workshops. Instructors explained that men prefer physical activities and competition. One respondent said, “sitting in a classroom is not something that older males look forward to,” and another said, “older men are more about doing and fixing than talking.” Regarding the EnhanceFitness (EF) program in particular, a common theme was that males would prefer other types of fitness activities. Respondents explained that men feel EF is not vigorous enough and emphasizes dancing too much.

A final theme that emerged was negative perceptions of health promotion programs. One respondent said, “the biggest barrier seems to be the attitude of older men towards the healthcare profession. They are less inclined to visit doctors and are not as proactive about their health as women.” One instructor shared that a man described the health promotion classes as “long-winded with a lot of common sense stuff.”

#### 3.2.3. Likelihood That Strategies Will Succeed in Increasing Male Participation

When presented with a list of strategies and asked to rate the likelihood that each strategy would increase male participation, all of the strategies were rated as likely to succeed by the majority of instructors. Over 90% of the instructors rated advertisements showing men engaging in health promotion and including pictures of single older males in promotional materials as likely to succeed (Table 3).

#### 3.2.4. Strategies Suggested to Increase Male Participation

Three main themes emerged from the open-ended question regarding strategies to improve male participation in health promotion programs. The most common theme among strategies suggested by respondents involved advertising and support for the HARC programs. Instructors suggested using local sports franchises and retired athletes to endorse the programs. They also suggested having male instructors and doctors promote the programs. Social media, television, and newspapers were suggested as important mediums for advertising older men participating in health promotion programs. Targeting advertisements where men congregate and actually offering programs where men congregate were also suggested to increase male participation.

The second theme discovered among suggested strategies was normalizing male participation in health promotion programs. The strategies to normalize male participation are closely linked with strategies to improve advertising and support for the programs. Including men in class advertisements and having recognized men in the community endorse male participation were suggested as strategies to “make attendance more acceptable or normal for older men.” Instructors also mentioned that offering men-only programs may help.

Another common theme that emerged deals with the content and perceptions of the EF program. Instructors suggested that the male participation in EF would be improved by using exercises that resemble circuit training, incorporating sports moves that both men and women could enjoy, and reducing the amount of dancing.

## 4. Discussion

People who engage in healthy lifestyle behaviors have a reduced risk for chronic disease morbidity and mortality [28]. Evidence-based health promotion programs have the potential to help participants adopt health behaviors to improve their wellbeing, but participation by males is suboptimal. Maximizing male participation in health promotion is an important goal for public health, because men are more likely than women to engage in most health risk behaviors, die at higher rates than women from the leading causes of death, and have shorter life spans than women [23, 29].

The current study enrolled HARC program coordinators and instructors who provided their perspectives on the barriers to recruiting older males in EBHP programs and suggested potential strategies to increase male participation. Coordinators and instructors are responsible for recruiting and engaging participants in EBHP, so their combined perspectives are valuable in understanding and improving male participation. The findings from the focus group and survey revealed that male gender roles, men's views of health promotion programs, preferences for other activities, and the low numbers of males in the classes are barriers to engaging males in health promotion programs. The findings from the survey supported most of the ideas reported in the focus group. Survey respondents did not, however, support the idea that women instructors are a barrier to male participation.

The findings from this study are in line with previous research which has reported that gender is associated with different health beliefs and behavior [30, 31]. From the perspective of the constructionist theory of gender, men and women think and act as they do because of socially constructed concepts about masculinity and femininity in their culture [32, 33]. The male gender role includes being independent, strong, and self-reliant and does not tend to include seeking care [23]. Applying this theory, men may avoid attending health promotion programs if participation in the program does not align with their concepts of masculinity.

Coordinators and instructors reported that men hold negative views of health promotion programs and the healthcare profession. This finding may reflect men's view of help-seeking in general. Researchers have reported that men with traditional beliefs about male gender roles have more negative attitudes about seeking help and are less willing to seek help [34]. Machismo, defined as strong masculine pride, has also been reported to affect help-seeking among Latino men and South Florida has a large Latino population [35]. Latino men who participated in a study of gender beliefs and prostate cancer screening indicated that machismo impedes preventive healthcare and screening [36]. The present study builds upon previous research by indicating that machismo acts as a barrier to male participation in EBHP programs as well.

A qualitative study of older British men's health needs indicates that although masculine culture has a strong influence on the way men think about health, men still wanted more health services and information, were open to discussing their healthcare needs, and were dissatisfied with masculine stereotypes in advertising and health information for men [25]. These findings emphasize the importance of acknowledging the influence of masculinity reported in the current study while remaining sensitive to the multitude of other factors, such as culture, socioeconomic status, ethnicity, and sexual orientation, which may influence men's needs and preferences related to health promotion programs.

Coordinators and instructors indicated that older men prefer alternatives to the current discussion-based activities provided in HARC programs. Previous research on masculinity and health beliefs supports the notion that men do not discuss health issues unless they are serious and solvable and that men are averse to disclosing vulnerability [37]. Gast and Peak reported that men are willing to discuss health concerns but that male-friendly health education programs are needed because men may not feel comfortable with the way services are currently delivered [38]. Men's Sheds represent a novel approach to enhancing the health of men in Australia by providing a male-friendly space and should be investigated for use with older men in the United States [39]. The Men's Shed is a large shed that allows men to come together to socialize and partake in meaningful activities such as woodwork or metalwork which help to provide a sense of connection and purpose that support health [39]. The Australian Men's Sheds do not ensure the provision of specific health information or skills needed by older adult men, but lessons learned from work with Men's Sheds about the activities and environments used to reach men could be applied to health promotion programs and recruiting strategies.

Coordinators and instructors expressed that females' outnumbering males was a barrier to participation. Carroll et al. found that homogenous group composition was important for creating a safe dynamic and supported the engagement of men in community-based health and physical activity promotion programs [40]. Saunders et al. also found that men were more likely to participate in male-only church-based programs and appreciated the opportunity to develop relationships with male peers and to discuss topics affecting them [41]. It is possible that male participation could be improved by offering workshops exclusively for men, but respondents in the current study also indicated that attending with spouses or partners could improve male participation. Staff delivering chronic disease and management programs in rural areas in Canada were interviewed in a qualitative study and also reported that spouses were important for encouraging male participation [42].

Other strategies suggested by coordinators and instructors relate to the participation barriers posed by gender roles. Instructors suggested that public support of the programs by male community leaders and advertisements that highlight male participation would help to normalize and increase male participation in the programs. Friedman et al. confirmed that using of mass media, partnering with businesses and churches, enlisting community leaders and role models as spokespeople, and culturally appropriate messages were seen by middle-aged and older African American men as important strategies for promoting physical activity programs [43].

The strategies described in the current study also emphasize addressing content and perceptions of EnhanceFitness to make it more appealing to men. Respondents did not explicitly suggest adapting the other evidence-based programs offered by the HARC, but the barriers they described point to program adaptation as an important strategy for meeting the needs of men.

### 4.1. Limitations

The main limitation of this study was that the older male participants and nonparticipants were not included in the study. Instructors and coordinator perspectives were examined to understand the barriers to recruiting and engaging older adult men in the region served by the HARC. Future research should examine male gender roles, perceptions of health promotion programs, and preferences among older males who participate and older males who decline the opportunity to participate in EBHP programs.

Another limitation of the study was the low response rate for instructors. Many HARC instructors taught on a volunteer basis and likely did not respond due to the transient nature of their positions as HARC instructors. Also, the majority of the study participants were female which introduces the potential for gender bias. However, analysis did not reveal gender differences in the percentage of instructors reporting agreement with the barriers or the percentage of instructors rating the likelihood of success of strategies. Finally, this study asked instructors and coordinators about EBHP programs in general but it will be important to investigate whether there are similarities across programs or if the factors influencing male participation differ by program.

## 5. Conclusion

This study provided novel information about barriers to older male participation in evidence-based health promotion programs and strategies to improve participation by older males. The barriers to male participation identified by program coordinators and instructors suggest that men feel outnumbered in health promotion programs, perceive participation as incompatible with the male gender role, and prefer other activities. Overall, the results of this study highlight a number of ways in which the EBHP programs are not well-suited to the needs of older men. The findings indicate that program planners could increase male participation in EBHP programs by tailoring recruiting strategies to men's gender-based perceptions regarding participation in health promotion, adapting health promotion programs to the beliefs and preferences of men, and improving advertising efforts. To advance men's health promotion, research should examine health promotion programs and recruiting strategies to ensure that they are meeting older men's needs and preferences.

## Figures and Tables

**Table 1:** Agreement with statements explaining why older males do not participate in HARC programs.

Statement	Agree	Disagree
Older males view exercise programs as aerobics classes for females.	77.6%	22.4%
Machismo or cultural beliefs prevent older men from participating in health promotion programs.	73.5%	26.5%
Older males think personal problems should not be discussed.	73.5%	26.5%
There are too many women in health promotion classes.	49.0%	51.0%
Older males feel that there is a negative stigma associated with participating in health promotion programs for older adults.	42.9%	57.1%
Older males believe that they will be perceived as old if they participate in a health promotion program.	30.6%	69.4%
Older males think that they will not be respected if they participate in a health promotion program.	22.5%	77.5%
Older males are not comfortable with female instructors.	18.4%	81.6%

*Note.* For all statements, the sample size was *n* = 49.

**Table 2:** Agreement with statements regarding men's beliefs and participation.

Statement	Agree	Disagree
Men are more likely to participate if they see other men in the group.	95.7%	4.3%
Men are more likely to participate with their spouse/partner.	87.2%	12.8%
Men view group exercise as a female activity.	76.6%	23.4%
Men are reluctant to attend organized health promotion activities.	66.0%	34.0%
Men believe they have little control over what happens to them in the aging process.	57.4%	42.6%
Male participants are not actively engaged in workshops.	57.4%	42.6%
Men think that seeking health advice makes them seem weak or dependent.	55.3%	44.7%
Men will stop coming to classes if they are the only male in the class.	55.3%	44.7%
Men feel awkward or embarrassed in an exercise or health education class.	51.1%	48.9%
Male participants think the material covered in the workshops is “silly” or too “touchy-feely.”	44.7%	55.3%
Male participants are likely to lose interest during a workshop session.	44.7%	55.3%
Men prefer male instructors to female instructors.	25.5%	74.5%

*Note.* For all statements, the sample size was *n* = 47.

**Table 3:** Likelihood of strategies successfully increasing male participation in HARC programs.

Strategy	Likely	Unlikely
Produce advertisements that show men engaging in health promotion programs.	95.7%	4.3%
Have pictures of single older males in program brochures, flyers, and posters (not just couples).	91.3%	8.7%
Get male community leaders to publicly support the program (e.g., church leaders, politicians, and community leaders as advocates).	89.1%	10.9%
Air public service announcements aimed at men.	89.1%	10.9%
Get well known public figures to endorse male participation.	87.0%	13.0%
Encourage women who register for classes to bring men.	82.6%	17.4%
Provide meaningful incentives (e.g., gift card to a store where men typically shop).	78.3%	21.7%
Go to barbershops.	63.0%	37.0%
Hire more male instructors.	60.9%	39.1%

*Note.* For all strategies, the sample size was *n* = 46.
